# Injury-induced Cavl-expressing cells at lesion rostral side play major roles in spinal cord regeneration

**DOI:** 10.1098/rsob.200304

**Published:** 2021-02-24

**Authors:** Chih-Wei Zeng, Yasuhiro Kamei, Shuji Shigenobu, Jin-Chuan Sheu, Huai-Jen Tsai

**Affiliations:** ^1^ Institute of Molecular and Cellular Biology, College of Life Science, National Taiwan University, Taipei 10617, Taiwan; ^2^ Liver Disease Prevention and Treatment Research Foundation, Taipei 10008, Taiwan; ^3^ Spectrography and Bioimaging Facility, National Institute for Basic Biology (NIBB), National Institutes of Natural Sciences (NINS), Okazaki 444-8585, Japan; ^4^ Department of Basic Biology, The Graduate University for Advanced Studies (SOKENDAI), Okazaki 444-8585, Japan; ^5^ Functional Genomics Facility, NIBB, NINS, Okazaki 444-8585, Japan; ^6^ Institute of Biomedical Sciences, Mackay Medical College, New Taipei City 25245, Taiwan; ^7^ Department of Life Science, Fu Jen Catholic University, New Taipei City 242062, Taiwan

**Keywords:** axon, motoneuron, neuronal regeneration, spinal cord injury, transgenic zebrafish, caveolin-1

## Abstract

The extent of cellular heterogeneity involved in neuronal regeneration after spinal cord injury (SCI) remains unclear. Therefore, we established stress-responsive transgenic zebrafish embryos with SCI. As a result, we found an SCI-induced cell population, termed SCI stress-responsive regenerating cells (SrRCs), essential for neuronal regeneration post-SCI. SrRCs were mostly composed of subtypes of radial glia (RGs-SrRCs) and neuron stem/progenitor cells (NSPCs-SrRCs) that are able to differentiate into neurons, and they formed a bridge across the lesion and connected with neighbouring undamaged motor neurons post-SCI. Compared to SrRCs at the caudal side of the SCI site (caudal-SrRCs), rostral-SrRCs participated more actively in neuronal regeneration. After RNA-seq analysis, we discovered that *caveolin 1* (*cav1*) was significantly upregulated in rostral-SrRCs and that *cav1* was responsible for the axonal regrowth and regenerative capability of rostral-SrRCs. Collectively, we define a specific SCI-induced cell population, SrRCs, involved in neuronal regeneration, demonstrate that rostral-SrRCs exhibit higher neuronal differentiation capability and prove that *cav1* is predominantly expressed in rostral-SrRCs, playing a major role in neuronal regeneration after SCI.

## Graphical abstract

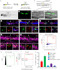

## 1. Introduction

Spinal cord injury (SCI) in mammals can cause irreversible loss-of-function because the capacity for regeneration is relatively low [1]. Cell therapy, in which exogenous cells are implanted to form a replacement neuronal population and (or) provide a growth-promoting microenvironment, is considered a promising strategy to clinically restore function following SCI. These therapeutic cells may be derived from adult [2] or fetal [3] embryonic stem cells, somatic neural stem/progenitor cells, or umbilical cord blood cells [4]. A combination of different cell types capable of participating in nerve regeneration, such as Schwann cells, olfactory ensheathing glial cells and neurotrophin-expressing fibroblasts [5–7], may also be applied. However, the efficacy of exogenous cell transplantation in clinical trials has been limited [8], suggesting that the necessary signalling factors and regenerative cell subtypes have yet to be clearly identified.

Amphibians are able to regenerate their spinal cord prior to metamorphosis. For example, when the spinal cord of a tadpole is transected, serotonergic axons can be observed crossing the lesion site, resulting in the restoration of tadpole swimming ability. However, they fail to undergo regenerative processes after SCI past metamorphosis [9]. Birds display a partial regeneration of the spinal cord at the larval stage, but they totally lose their regeneration capability over the course of maturation [10]. In humans, SCI is almost an irreversible process because regenerative capability is limited [1,11]. However, mice retain their spinal cord's regenerative capability until a few days after birth, after which this ability decreases as development progresses [12]. Interestingly, Reimer *et al*. [13] reported newly formed spinal motor neurons and restoration of swimming in zebrafish following spinal cord transection [13]. Hui *et al*. [14] reported that glial cells could be induced to contribute to neuronal regeneration in zebrafish following crush injury. Briona & Dorsky [15] also demonstrated that *dbx1a*-green fluorescence protein (GFP)-expression radial glia cells could dedifferentiate into stem cells and give rise to spinal interneurons within 5 days following spinal cord transection in zebrafish [16]. Mokalled *et al*. [17] found that connective tissue growth factor a (CTGFa)-expressing glial cells could form glial bridges to promote spinal cord regeneration.

Many strategies have been attempted to identify cell types that may contribute to neural regeneration following SCI. One strategy is to use a specific antibody to identify a particular cell type, such as SOX2-positive neural progenitor cells, A2B5-positive astrocyte/glial progenitor cells and NG2-positive oligodendrocyte/Schwann cell-like progenitors. Another strategy is to employ a transgenic line expressing a particular gene driven by cell-type-specific promoter, such as *Tg(her4.1:mCherry,Cre-ERT2)*, which labels her4.1-positive ventricular radial glial progenitor cells [18], *Tg(−3.5dbx1a:EGFP),* which label neuronal cells during embryogenesis [16] or *Tg(−8.4neurog1:GFP)*, which labels young migrating neural progenitor cells [19]. However, neuronal regeneration seems to require multiple cell types or subtypes. For example, Goldshmit *et al*. [20] observed glial fibrillary acid protein (GFAP)-positive (+) cells around the central canal in zebrafish at 3 days post-injury (dpi), as well as several unknown cell populations at 5 and 10 dpi [20]. These glial cells were able to migrate to the lesion site and form bridges by the third week, resulting in the complete recovery of nerves by the fifth week post-SCI [20]. Hui *et al*. [16] and Zeng *et al*. [21] reported that neural stem cells, differentiated stem-like cells and other unidentified cells are cell populations involved in recovery from SCI [16,21]. Furthermore, different methods of inducing SCI elicit the participation of different cell populations to play roles in neuronal regeneration [21,22]. Therefore, using a specific protein marker, or a transgenic line driven by a tissue- or cell-specific promoter, does not capture the whole galaxy of cell populations involved in neuronal regeneration. Consequently, it remains unknown if different cell types give rise to specific subtype-cell populations that converge at the time of mechanically induced SCI to perform neuronal regeneration and functional recovery. SCI would certainly result in endoplasmic reticulum (ER) stress to cells located at the injury site.

It is easy to hypothesize that SCI would result in ER stress to cells located at the injury site. However, such injury is compounded, even at the initial stage, by the induction of unfolded protein response (UPR), during which eukaryotic initiation factor eIF2*α* is phosphorylated to reduce global protein synthesis [23]. At the same time, C/EBP homologous transcription factor protein (CHOP) is activated by inositol-requiring protein 1 and ATF6 transcription factor [23], resulting in extensive translation of *chop* mRNAs. Under normal conditions, though, *chop* translation is inhibited by the upstream open reading frame (uORF) at the 5'-untranslated region (UTR) of *chop* mRNA (*uORF^chop^*) [24]. On the contrary, under the stress condition, UPR regulates downstream gene expression to overcome ER stress before apoptosis of affected cells [25]. Consequently, *uORF^chop^*-mediated translation inhibition is suppressed, resulting in the translation of CHOP protein. Such increased CHOP protein production promotes the survival of neuronal cells against hypoxia-stress-induced death [26]. Therefore, CHOP is a useful protein marking the presence of ER stress within cells. Lee *et al*. [27] generated a zebrafish transgenic line *huORFZ* harbouring a human *uORF^chop^* (*huORF^chop^*) motif to inhibit the translation of downstream GFP reporter in the absence of stress [27]. However, GFP is exclusively expressed in the CNS of *huORFZ* embryos encountering such stressors as heat-shock [27] and hypoxia [21]. For example, when *huORFZ* embryos were exposed to hypoxia, GFP was only expressed by a specific subtype-cell population, termed hypoxia-responsive recovering cells (HrRCs), in the CNS. These HrRCs consist of multiple cell types and subtypes that contribute to neuronal regeneration after hypoxia. More importantly, such HrRCs can survive and play rescue and regenerative roles in the post-lesion microenvironment [21]. In this study, we used a mechanical method to cause SCI stress in zebrafish. We speculate the existence of a cell or subtype cell population in the spinal cord able to respond to ER stress in a manner similar to hypoxic stress in which some neurons survive to play a regenerative role in the same type of microenvironment. Therefore, the *huORFZ* line may also be an excellent model to study the characteristics of major cell subtypes participating in neuronal regeneration after mechanically induced SCI. Additionally, some studies have found that cells located at the side close to the head part (rostral side) and cells close to the tail part (caudal side) of the SCI contribute equally to neuronal regeneration [16,28]. On the other hand, Briona & Dorsky [15] reported that the regenerative response appeared to be delayed and muted on the caudal side. Therefore, no consensus has, thus far, been clearly reached in the matter of rostral versus caudal cell biology and function in the context of neuronal regeneration after SCI.

Thus, in the present study, we attempted to resolve key questions and competing hypotheses, as noted above, by identifying (i) any specific cell population, in turn consisting of different subtype cells involved in neuronal regeneration following SCI stress, as well as the composition and proportion of cell types and subtypes belonging to such a specific subtype-cell population; (ii) the biological characteristics and functions of such a specific subtype-cell population relative to rostral versus caudal sides of SCI; and (iii) any major gene involved in differentiating rostral versus caudal cells in the regeneration processes. To address these issues, we subjected *huORFZ* embryos to mechanical SCI and identified a specific subtype-cell population, termed as SCI stress-responsive regenerating cells (SrRCs). By their collective complex of subtype cells, SrRCs include such major subtypes as radial glia (RGs-SrRCs) and neural stem/progenitor cells (NSPCs-SrRCs) that are entirely resistant to SCI stress and able to differentiate in order to play a major role in axonal regeneration. Moreover, compared to caudal-SrRCs, rostral-SrRCs appear to play a distinct and preeminent role in regenerative activity following SCI.

## 2. Results

### 2.1. A specific subtype-cell population that resisted spinal cord injury stress and survived it appeared at both sides of the spinal cord injury site in *huORFZ* transgenic embryos

**Figure 1.**
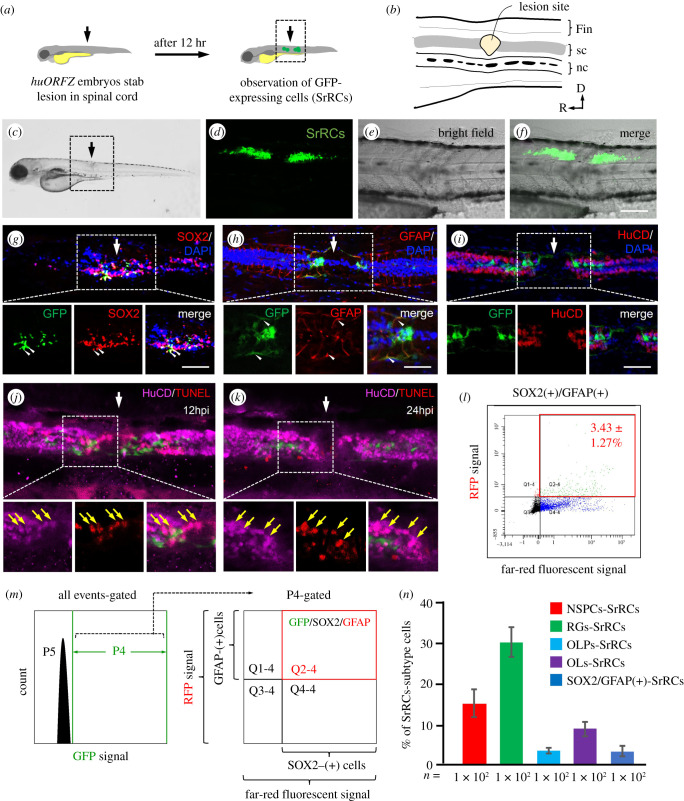
A specific subtype-cell population found in spinal cord of zebrafish larvae following SCI was identified. (*a*) Schematic diagram of the experiments following SCI. Arrow indicates the spinal cord (sc) injury site. (*b*) The enlarged image of SCI site shown in the box in (*a*). nc: notochord. (*c*) Zebrafish embryos from transgenic line *huORFZ* at 72 hours-post-fertilization (hpf) were treated with SCI, as indicated by the arrow. Enlarged image of the box area indicated in (*c*) was observed under microscopy: (*d*) fluorescence, (*e*) bright-field and (*f*) merged images, in which GFP-expressing cells (SrRCs) were apparent on both sides of the SCI site at 12 h post-injury. Scale bar indicates 50 µm. (*g*–*i*) Immunostaining identified cell types among the subtype cell population of SrRCs. White arrow indicates the location of SCI site. Red fluorescence was specifically labelled as (*g*) SOX2, (*h*) GFAP and (*i*) HuCD. Yellow fluorescence signal indicated that GFP-expressing SrRCs overlapped target protein labelled by red fluorescence signal, as indicated by yellow arrows. Scale bar indicates 15 µm. (*j*,*k*) TUNEL assay identified apoptotic cells by red fluorescence signal. Neurons were identified by marker HuCD labelled with far-red fluorescence signal. The apoptotic neurons appeared in pink colour. (*l*,*m*) After FACS, the isolated SrRCs were immunostained with antibodies against SOX2 and GFAP (P5: background; P4: cells expressing GFP signal; Q2–4: GFP(+) cells co-expressing SOX2-infrared light signal and GFAP-RFP signal). (*n*) Calculating the ratio of each cell type among GFP-expressing cell populations isolated by FACS. Error bars indicate s.e.m.

When zebrafish embryos from transgenic line *huORFZ* were subjected to crush injury at the lateral side of the spinal cord to cause mechanical SCI (figure 1*a*,*b*), a specific subtype-cell population on both sides of the injury site expressed GFP signal at 12 h post-injury (hpi). We termed these GFP-expressing cells as SCI stress-responsive regenerating cells (SrRCs) based on their resistance and responsiveness to SCI stress (figure 1*c*,*f*). To differentiate the cell types among SrRCs, we employed fluorescence-activated cell sorting (FACS) and cell immunostaining to identify NSPCs and RGs and found that most SrRCs overlapped SOX2-red fluorescence protein (RFP)-labelled NSPC cells (figure 1*g*) and GFAP-RFP-labelled RGs (figure 1*h*), but did not overlap Hu antigen C and D proteins (HuCD)-RFP-labelled neurons (figure 1*i*,*k*), indicating that SrRCs were not neurons. Furthermore, we demonstrated that SrRCs were composed of RGs at 31.92 ± 3.83%, NSPCs at 15.90 ± 3.6%, oligodendrocyte progenitor cells (OLPs) at 10.15 ± 1.45% and oligodendrocytes (OLs) at 3.51 ± 0.74% (figure 1*n*). A limited number of SrRCs belonging to the NSPCs cell type (NSPC-SrRCs subtype) co-expressed double signals of GFAP-RFP and SOX2-infrared with a ratio of 3.43 ± 1.27% (figure 1*l,m*).

When the terminal deoxynucleotidyl transferase dUTP nick end labeling (TUNEL) assay was performed, GFP-negative cells (non-SrRCs) were found to be apoptotic since they were labelled with the apoptotic TUNEL-RFP signal, whereas GFP-expressing SrRCs were not (figure 1*j*,*k*). Importantly, if HuCD-far-red fluorescence signal was used to label neurons, we found that the apoptotic TUNEL-RFP signals were also overlapped with HuCD-far-red-labelled neurons, resulting in a pink colour in the SCI-embryos at 12 (figure 1*j*) and 24 hpi (figure 1*k*), indicating that neurons at the SCI site were non-SrRCs since they were apoptotic. Taken together, we suggested that SrRCs were resistant to SCI stress and might contribute to neuronal regeneration at the lesion site after SCI.

### 2.2. NSPCs-SrRCs subtype connected with motor neurons through axonal elongation, while RGs-SrRCs subtype formed a cellular bridge during neuronal regeneration

SrRCs were apparent at both sides of the SCI site. Rostral-SrRCs comprised one group that distributed at the injury side close to the head, whereas caudal-SrRCs distributed at the injury side close to the tail. At 48 hpi, we observed that SrRCs could promote wound healing, in which elongated axons of both rostral- and caudal-SrRCs connected with neighbouring undamaged motor neurons located at both sides of the injury site during neuronal regeneration (electronic supplementary material, figure S1A). To determine which particular cell subtype among NSPCs is responsible for initiating such connections with motor neurons, we collected *huORFZ* larvae after SCI, followed by whole-mount immunohistochemistry (IHC) for SOX2, Acetyl-Tubulin (AT), and GFAP. Results showed that only SOX2(+)/GFP(+) subtype cells that were NSPCs-SrRCs, but not SOX2(+)/GFP(–) subtype cells, could initiate a connection with motor neurons on both sides of the SCI site at 24 hpi (electronic supplementary material, figure S1B). In electronic supplementary material, figure S1B, we show that only SOX2(+)/GFP(+) subtype cells, which were NSPCs-SrRCs, but not SOX2(+)/GFP(−) subtype cells, could connect with motoneurons on both sides, caudal and rostral, of the SCI site at 24 hpi. Both rostral- and caudal-NSPCs-SrRCs started to extend axons at 36 hpi. Similarly, only GFAP(+)/GFP(+) subtype cells that were RGs-SrRCs, but not GFAP(+)/GFP(−) subtype cells, started to form the glial bridge between the two sides of SCI site at 24 hpi (electronic supplementary material, figure S1C). At 36 and 48 hpi, these RGs-SrRCs continued their bridge-building activity across the SCI site (electronic supplementary material, figure S1C). Therefore, we reasoned that NSPCs- and RGs-SrRCs cell subtypes, both of which are highly responsive to SCI stress and able to survive it, are the two main constituents able to initiate spinal cord regeneration in zebrafish after SCI. Using ImageJ to analyse IHC images, we identified the location and quantified the number of NSPCs- and RGs-SrRCs that had distributed around the SCI site. As described in Materials and Methods, the rostral side of the SCI site could be divided into the blastemal area (BL), the neighbouring proximal (PL) area and the rostral (RL) area. Results showed that NSPCs-SrRCs were mostly located at the RL (0.12 ± 0.04 pixels) and PL (0.22 ± 0.03 pixels) areas at 24 hpi and that they mostly located at the PL (0.37 ± 0.06 pixels) and BL (0.28 ± 0.04 pixels) areas at 48 hpi (electronic supplementary material, figure S1B,D). As recovery time increased, the number of NSPCs-SrRCs correspondingly increased. Moreover, while cell bodies moved toward the injury site to participate in neuronal regeneration (electronic supplementary material, figure S1B), we noticed that their elongated axons had connected with undamaged motor neurons on the other side of the lesion site (electronic supplementary material, figure S1B). For example, at 24 hpi, most NSPCs-SrRCs were distributed at the RL area where undamaged motor neurons were located, but only a limited number of NSPCs-SrRCs were distributed at the BL nearest the SCI site (electronic supplementary material, figure S1B,D). However, at 48 hpi, most NSPCs-SrRCs were distributed at the PL and BL areas, but only a limited number of NSPCs-SrRCs were distributed at the RL (electronic supplementary material, figure S1B,D). On the other hand, although RGs-SrRCs were predominantly and increasingly distributed at the BL area (0.44 ± 0.05 pixels) from 24 through 48 hpi, no RGs-SrRCs had distributed to the PL area (electronic supplementary material, figure S1C,D). These results were consistent with IHC analysis, which demonstrated that the elongated axons of NSPCs-SrRCs initially connected with motor neurons at the RL area during the early stage of regeneration (electronic supplementary material, figure S1C), while RGs-SrRCs formed glial bridges (electronic supplementary material, figure S1E,G) and they mostly distributed at the BL area.

### 2.3. RGs-SrRCs subtype made a predominant contribution to neuronal regeneration

To determine if a specific subtype among RG cells plays a major role in the neuronal regeneration of SCI-embryos, we employed a double-transgenic line established by crossing line Tg(*gfap*:tomato) and line *huORFZ*, showing RFP-marked GFAP cells and GFP-marked SrRCs after SCI (electronic supplementary material, figure S2A). The embryos from these double-transgenic line were treated with SCI, followed by comparing swimming performance between RFP(+)-ablated larvae and RFP(+)/GFP(+)-double-ablated larvae. Both sides of the SCI site at 12 hpi displayed GFP-expressing SrRCs and yellow fluorescence-expressing RGs-SrRCs in the SCI-treated the larvae (electronic supplementary material, figure S2B). After infrared (IR) ablation, the fluorescence signals disappeared on the target cells (electronic supplementary material, figure S2B), suggesting that the application of IR-laser had ablated specific fluorescent cells, as we demonstrated previously in electronic supplementary material figure S1b. Analysis of swimming performance demonstrated that the swimming distance of RGs-non-SrRCs-ablated larvae (RFP(+)-ablated larvae) was 64.4% further than that of RGs-SrRCs-ablated larvae (RFP(+)/GFP(+)-double-ablated larvae) at 72 h-post-ablation (electronic supplementary material, figure S2C,D). Among the RGs cell type, this result suggested that the RGs-SrRCs subtype plays a more predominant role in neuronal regeneration after SCI compared to RGs-non-SrRCs.

### 2.4. *Connective tissue growth factor* (*ctgf*) mRNA and encoded CTGF protein were expressed in SrRCs

We found that RGs formed primary glial bridging. Importantly, it has been reported that a matricellular protein, CTGF, expressed in RGs is necessary for glial bridging during spinal cord regeneration [17]. Thus, we studied whether CTGF is also expressed in SrRCs during neuronal regeneration after SCI. Results showed that *ctgf* mRNA was present on both sides of the SCI site at 30 hpi (electronic supplementary material, figure S2E). The level of *ctgf* mRNA expressed in rostral-SrRCs was much higher than that in caudal-SrRCs (electronic supplementary material, figure S2E). Moreover, Western blot analysis demonstrated that CTGF was present in both rostral- and caudal-SrRCs (electronic supplementary material, figure S2H). However, the amount of CTGF in rostral-SrRCs was significantly greater than that observed in caudal-SrRCs at 24, 36 and 48 hpi (electronic supplementary material, figure S2F,G). We also noticed that the highest level of CTGF expression was at 36 hpi (electronic supplementary material, figure S2H), corresponding with the time point at which most RGs-SrRCs were observed to form glial bridges (electronic supplementary material, figure S1). Therefore, we concluded that CTGF has a high level of expression in rostral-SrRCs, further supporting that rostral-SrRCs are, functionally, preeminent players in the neuronal regeneration of SCI-larvae.

### 2.5. Some spinal cord injury-induced SrRCs differentiated into neurons

**Figure 2.**
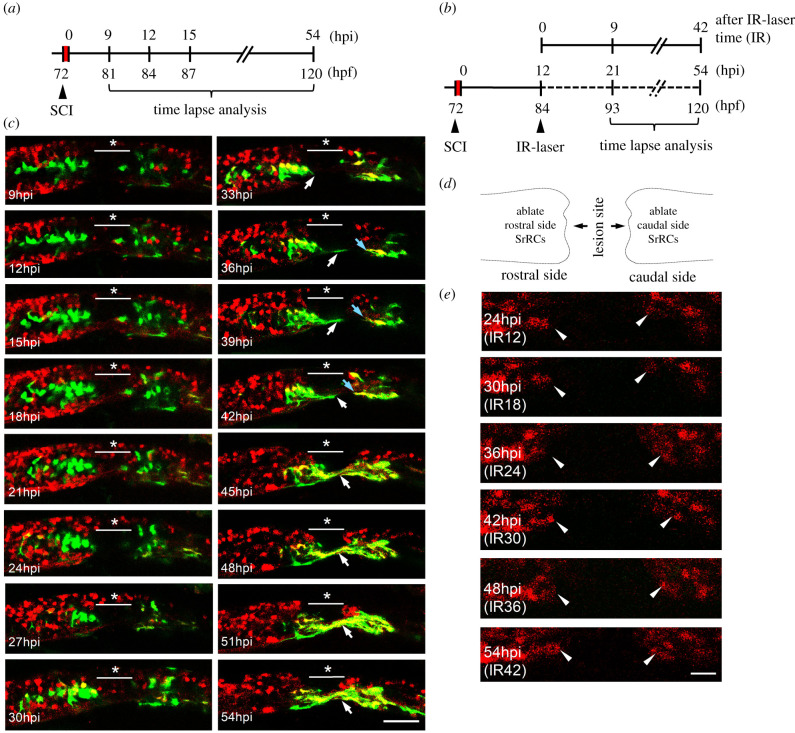
Using an *in vivo* system to confirm that SCI-induced SrRCs played a role in neuronal regeneration. (*a*) Schematic diagram of the timetable designed for observing the neuronal regeneration of SCI-larva. hpi: hours-post-injury; hpf: hours-post-fertilization. (*b*) Schematic diagram of the time table designed for observing the neuronal regeneration of SCI-larvae after infrared (IR)-LEGO laser (IR) treatment which was used to ablate their GFP-expressing cells. (*c*) Double-transgenic embryos derived from line *Tg*(*huc*-*DsRed*), crossed with line *huORFZ*, were used to dynamically trace neural differentiation and regeneration following SCI by confocal time-lapse photography. The underscored stars indicate the location where SCI was performed, while white arrows point to cells differentiated into neurons and their extended axons at the series time point indicated. (*d*) Diagram illustrated that SrRCs located at both sides (rostral and caudal) of SCI site were ablated. (*e*) Tracing neuronal differentiation and regeneration at series time point, as indicated, after both sides of SrRCs of SCI-larvae were ablated simultaneously. Photos were taken from lateral view in which dorsal side was upward, while rostral side was at left. Arrowheads indicate outgrowing neurites. Scale bar at right bottom was presented as 10 µm.

To further confirm that some SCI-induced SrRCs could differentiate into neurons, we performed an *in vivo* experiment by employing zebrafish double-transgenic embryos derived from the transgenic line *huORFZ* crossed with Tg(*huc:dsRed*), in which the HuC protein is tagged with red fluorescence protein (RFP; dsRed). Mechanical SCI was performed on these hybrid embryos, followed by tracing the SrRCs to determine whether they could differentiate into neurons and play a regenerative role post-SCI (figure 2*a*). No extended axons were observed at two clusters of SrRCs that were apparent at both sides of SCI from 9 to 30 hpi (figure 2*c*). However, some SrRCs started to present yellow fluorescence owing to their expression of the red fluorescent HuC protein at 30 hpi onset (figure 2*c*, 30–54 hpi), indicating that some SrRCs had begun to differentiate into early stage neurons. At 33 hpi, axons that developed from neurons started to extend. At 45 hpi, two sides of extended axons had synapsed with each other across the SCI site (figure 2*c*), suggesting that SrRCs at both sides of SCI lesion were able to differentiate into neurons, followed by the synapsis of their extended axons during axonal regeneration. More importantly, when we traced several single cells from the SrRCs subtype, no expression of early neuron marker in cells was observed until 27 hpi (electronic supplementary material, figure S3), suggesting that 30 hpi was the starting point at which SrRCs could differentiate into neurons.

To confirm the contribution of the SrRCs to the recovery of neurons, we employed IR laser illumination to ablate GFP-expressing SrRCs. When the SrRCs of SCI-embryos derived from the double-transgenic line, line *huORFZ* crossed with line *Tg*(*huc*-*DsRed*), were ablated, no HuC-red fluorescence signal was apparent from 30 to 51 hpi (figure 2*b–e*), suggesting that no neurons had been newly generated to process regeneration after SCI, even though these neurons had already developed at 51 hpi, unlike SCI-embryos that started to differentiate into neurons at 30 hpi, as shown above.

### 2.6. SrRCs played an important function in neuronal regeneration after spinal cord injury

To assess whether SrRCs contribute to neuronal regeneration and functional recovery after SCI, we ablated these cells by infrared (IR) laser illumination following lateral-side SCI at 72 hpf and evaluated the effects on swimming. No GFP signal was detected from 12 h post-IR treatment (IR12) to IR72 (electronic supplementary material, figure S4A). Yet, SrRCs were completely absent in the SCI-treated *huORFZ* larvae as evidenced by 4′,6-diamidino-2-phenylindole (DAPI) nuclear staining (electronic supplementary material, figure S4B). Since the GFP signal did not reappear during IR12 to 72, we confirmed that IR laser was able to specifically ablate GFP-expressing SrRCs, quite apart from any photobleaching effects. First, after IR ablation for 72 h (IR72), we found at least 80% of larvae able to swim among positive (neither SCI nor IR-laser), negative (SCI-only) and sham (SCI and ablation of GFP-negative subtype; non-SrRCs-ablation) controls, while less than one-third of larvae could swim in the SCI and SrRCs-ablated groups (electronic supplementary material, figure S5A,B), suggesting that SrRCs might be tightly related to neuronal regeneration after SCI. Second, we analysed the swimming distance and route. Results showed that the swimming performance of larvae in the negative and sham controls was only slightly inferior to that of positive control; however, the SCI- and SrRCs-ablated larvae displayed the poorest performance (electronic supplementary material, figure S5C,D; S6), suggesting that the presence of SrRCs is necessary for functional recovery of neuronal regeneration. Third, we analysed the C-bend angle of the trunk of larvae stimulated by the touch-evoked response. Results demonstrated that the C-bend angle of larvae displayed a slight difference between the sham group and two controls; however, the C-bend angle of SCI-larvae treated with SrRCs-ablation was relatively small, exhibiting a significant decrease compared to that of the other three groups (electronic supplementary material, figure S5E,G and movie 1), suggesting blockage of neuronal transmission in the absence of SrRCs. Taken together, we hypothesize that SrRCs play major roles in neuronal regeneration, providing normal neuronal transmission to allow the SCI-larvae to recover their locomotion.

### 2.7. Rostral- and caudal-SrRCs differed in terms of cell characteristics and functions relative to neuronal regeneration after spinal cord injury

**Figure 3.**
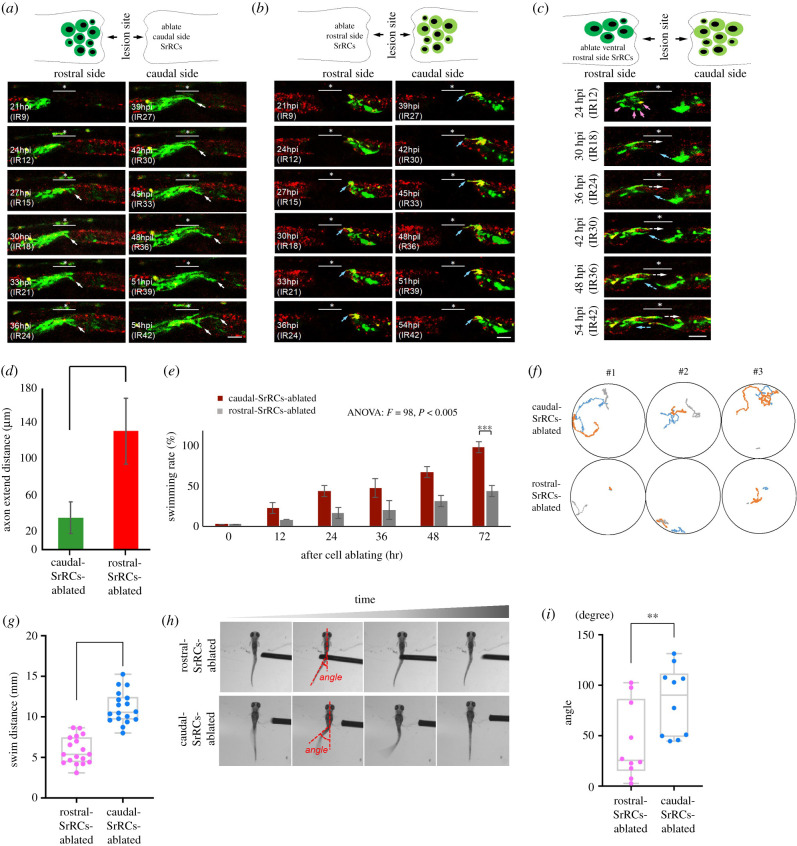
Rostral-SrRCs in the spinal cord of SCI-treated zebrafish larvae can prominently enhance functional recovery during neuronal regeneration. Double-transgenic embryos derived from transgenic line Tg(*huc-dsRed*), crossed with line *huORFZ*, were used to trace neural differentiation and regeneration following SCI by confocal time-lapse photography. (*a–c*) The underscore stars indicate the SCI sites, while white arrows point to cells differentiated into neurons and their extended axons. Dynamic traces of neural differentiation and regeneration of SCI-treated larvae upon termination of IR-Laser ablation (IR) for serial hours, as indicated, when (*a*) caudal-SrRCs, (*b*) rostral-SrRCs and (*c*) rostral-SrRCs on the ventral side were ablated. (*d*) Statistical analysis of the average of the axonal extended distance (μm) obtained from 10 larvae in each group after IR-laser ablation of caudal- or rostral-SrRCs of SCI-larvae. Unpaired *t*-test was used to perform statistical analysis of significant difference between two groups (***p < 0.001). (*e*) Statistical analysis of swimming recovery rates of SCI-treated larvae upon termination of IR-Laser treatment at IR0 (starting time) through IR72 (*t*-test: ***p < 0.001). (*f*) Using a high-speed camera system to capture the swimming route of SrRCs-ablated SCI-larvae at IR72. Numbers #1, #2 and #3 indicate three individuals out of six larvae in each group. Each one was recorded three times (as also referenced in electronic supplementary material, figure S7). (*g*) Comparison of swimming distance of larvae between two groups. Swimming distance (mm) was represented as overall mean for each group calculated from the total mean of 18 larvae. The average value for each larva was based on three independent trials. (Two-way ANOVA with Bonferroni multiple comparisons test, ***p < 0.005; *t-*test: ***p < 0.001). (*h*) After touch-evoked response, photos were taken to calculate C-band angle, which was the angle between the centre of gravity and the tail. (*i*) Statistical analysis of the overall average of the C-bend degrees calculated from 10 larvae in each group. Unpaired *t*-test showed that degree was significantly different between these two groups (**p < 0.01). Error bars indicate s.e.m.

A subset of SrRCs is found on each side of the SCI lesion, termed as rostral- (close to head) and caudal-SrRCs (close to tail). We noticed that axons extended from neurons differentiated from rostral-SrRCs were much more abundant and longer compared to axons from caudal-SrRCs (figure 2*c*; white arrow versus blue arrow), suggesting that rostral- and caudal-SrRCs might differ in terms of cell characteristics and functions involved in processing neuronal regeneration after SCI. To confirm this hypothesis, we separately ablated rostral- and caudal-SrRCs of SCI-larvae by IR laser illumination and observed the dynamics of neuronal regeneration during 21–54 hpi (equivalent to 9–42 h post-IR treatment; IR9-42). Neurons that differentiated from rostral-SrRCs began to extend axons at 27 hpi (IR15) upon ablation of caudal-SrRCs, continuously growing longer up to 54 hpi (IR42) (figure 3*a*). By contrast, when rostral-SrRCs were ablated, the extension of axons from caudal-SrRCs also began at 27 hpi, but did not grow longer, even though embryos had already substantially developed by 54 hpi (figure 3*b*). Moreover, axons that had begun to extend from caudal-SrRCs at 39 hpi were retracted at 54 hpi (figure 3*b*). Interestingly, when rostral-SrRCs at the ventral side were ablated, axons from caudal-SrRCs exclusively extended toward the dorsal side where rostral-SrRCs remained intact (figure 3*c*). After quantification, the results also showed that rostral-SrRCs continuously extended their axons until 54 hpi (IR42) when caudal-SrRCs were ablated. By contrast, when rostral-SrRCs were ablated, caudal-SrRCs did not extend axons, even though the embryos had developed at 54 hpi (figure 3*d*). This line of evidence suggests that axonal development from caudal-SrRCs depends on the presence of rostral-SrRCs.

Next, we ablated either rostral-SrRCs, or caudal-SrRCs, separately and examined the swimming performance of ablated larvae. Results demonstrated that significantly fewer larvae were able to swim in the rostral-SrRCs-ablated group compared to larvae in the caudal-SrRCs-ablated group at IR72 (figure 3*e*). The caudal-SrRCs-ablated SCI-larvae displayed longer swimming distance compared to the rostral-SrRCs-ablated SCI-larvae (figure 3*f*,*g* and electronic supplementary material, figure S7). Additionally, the C-bend angle of the trunk in rostral-SrRC-ablated larvae was significantly wider than that of caudal-SrRC-ablated larvae (figure 3*h,i* and electronic supplementary material, movie S2).

Since ablation of rostral-SrRCs hinders the swimming distance of SCI-larvae to a greater degree than ablation from caudal-SrRCs, it can be concluded that the functional recovery from SCI is impaired to a greater extent by rostral-SrRCs-ablation than by caudal-SrRCs-ablation, strongly suggesting that the cell characteristics and functions of rostral-SrRCs are different from those of caudal-SrRCs.

### 2.8. Rostral-SrRCs showed significantly higher neuronal differentiation capability compared to that of caudal-SrRCs during neuronal regeneration after spinal cord injury

**Figure 4.**
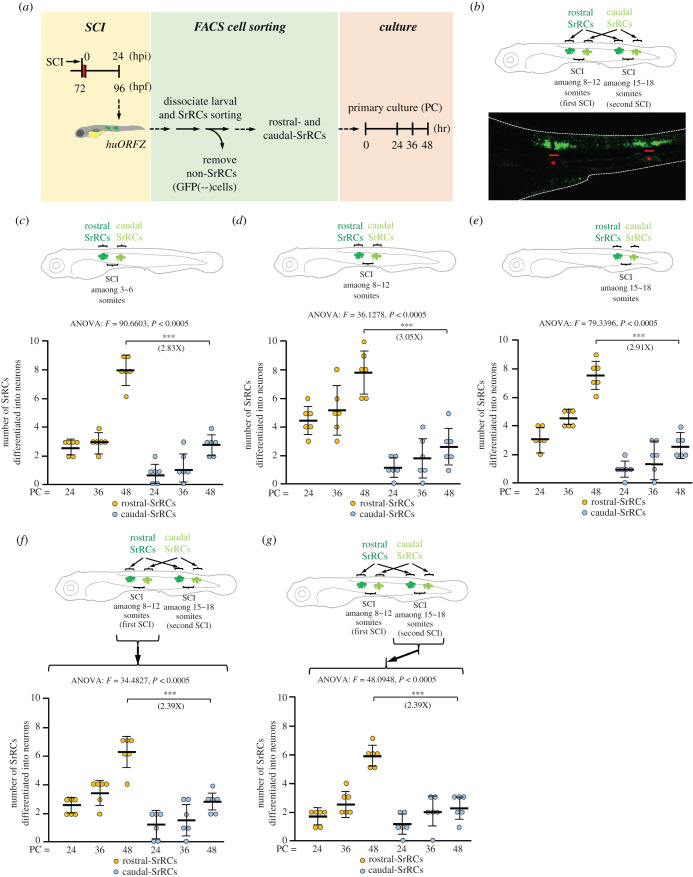
Assessment of neuronal differentiation capability showed that rostral-SrRCs achieved a higher fold increase than caudal-SrRCs during neuronal regeneration following SCI. (*a*) Schematic diagram illustrating the experimental design. (*b*) SCI was performed twice simultaneously at 8–12 and 15–18 somites. As demonstrated in the lower panel, a cluster of GFP-expressing SrRCs appeared at both sides of each SCI site, as observed under confocal microscopy. Stars combined with underscore indicate SCI sites. (*c–e*) Illustration of experimental design by performing single SCI at (*c*) 3–6, (*d*) 8–12 and (*e*) 15–18 somites. The numbers of collected rostral- and caudal-SrRCs able to differentiate into neurons after culturing were calculated separately, and the cell numbers after 48 h culture were compared by the increase in fold. (*f*,*g*) Illustration of experimental design by performing SCI simultaneously at 8–12 (the first SCI) and 15–18 somites (the second SCI). The numbers of collected rostral- and caudal-SrRCs from (*f*) the first 8–12 somite-SCI and (*g*) the second 15–18 somite-SCI sites able to differentiate into neurons after culturing were calculated separately, and cell numbers after 48 h culture were compared by the increase in fold. Two-way ANOVA with Bonferroni multiple comparisons test, ***p < 0.005; *t*-test: ***p < 0.001; each *F* value was also indicated. Error bars indicate s.e.m.

To know whether the regenerative capability of rostral-SrRCs depends on the location of the SCI site, we performed SCI at three different locations (3–6, 8–12 and 15–18 somites), collected rostral-SrRCs and caudal-SrRCs separately, and then cultured and evaluated for neuronal differentiation capability (NDC; figure 4*a*). For the 3–6-somite-SCI-embryos, the percentages of rostral-SrRCs that differentiated into neurons were 2.67 ± 0.52, 3.00 ± 0.63 and 8.00 ± 1.10% for 24, 36 and 48 h (electronic supplementary material, movie S3) culture, respectively, whereas the percentages of caudal-SrRCs that differentiated into neurons were 0.80 ± 0.75, 1.33 ± 1.03 and 2.83 ± 0.69% for 24, 36 and 48 h (electronic supplementary material, movie S4) culture, respectively (figure 4*c*), indicating that the NDC of rostral-SrRCs was 2.83-fold higher than that of caudal-SrRCs after 48 h culture. For the 8–12-somite-SCI-embryos, 4.50 ± 1.05, 5.33 ± 1.75 and 7.17 ± 2.32% of rostral-SrRCs differentiated into neurons for 24, 36 and 48 h culture, respectively, whereas 1.17 ± 0.75, 1.83 ± 1.47 and 2.39 ± 1.37% of caudal-SrRCs differentiated into neurons for 24, 36 and 48 h culture, respectively (figure 4*d*), indicating that the NDC of rostral-SrRCs was 3.05-fold higher than that of caudal-SrRCs after 48 h culture. Finally, for the 15–18-somites-SCI-embryos, 1.17 ± 0.75, 1.83 ± 1.47 and 2.39 ± 1.37% of rostral-SrRCs differentiated into neurons for 24, 36 and 48 h culture, respectively, whereas 1.00 ± 0.63, 1.83 ± 1.17 and 2.58 ± 0.73% of caudal-SrRCs differentiated into neurons for 24, 36 and 48 h culture, respectively (figure 4*e*), indicating, again, that the NDC of rostral-SrRCs was higher this time by 2.91-fold compared to that of caudal-SrRCs after 48 h culture. Collectively, we suggest that the higher NDC of rostral-SrRCs, compared to that of caudal-SrRCs, could not be attributed to the locus of SCI lesion.

It could be speculated that rostral-SrRCs gain an advantage in maintaining their predominant role in neuronal repair by the fact that some neurotransmitters are transmitted longitudinally from head to tail such as glycine [29], dopamine [30] and serotonin [31]. To make this determination, we performed a simultaneous double-SCI on larvae. In the first, SCI at 8–12 somites was close to head, while in the second, SCI at 15–18 somites was close to tail (figure 4*b*). Results showed that 2.50 ± 0.55, 3.50 ± 0.84 and 6.17 ± 1.17% of cells could differentiate into neurons from rostral-SrRCs isolated from 8–12-somite-SCI-embryos after culturing for 24, 36 and 48 h, respectively, whereas 1.18 ± 0.98, 1.67 ± 1.21 and 2.58 ± 0.73% of caudal-SrRCs from 8–12-somite-SCI-embryos could differentiate into neurons after culturing for 24, 36 and 48 h, respectively (figure 4*f*), indicating that the NDC of rostral-SrRCs was 2.39-fold higher than that of caudal-SrRCs in the first SCI at 8–12 somites after 48 h culture. Making the supposition that longitudinal transmission of neurotransmitters was interrupted by the first SCI lesion at 8–12 somites, we went continued on and found that the NDC of rostral-SrRCs in the second SCI at 15–18 somites was still 2.63-fold higher than that of caudal-SrRCs at the second SCI after 48 h culture (figure 4*g*), irrespective of injury site at the front 8–12 somites, suggesting that the higher NDC of rostral-SrRCs was unrelated to the transmitted direction of neuronal factors in the spinal cord. Rather, the higher NDC must be a unique characteristic of rostral-SrRCs.

### 2.9. Transplantation of rostral-SrRCs could effectively improve spinal cord regeneration in SCI-adult zebrafish

**Figure 5.**
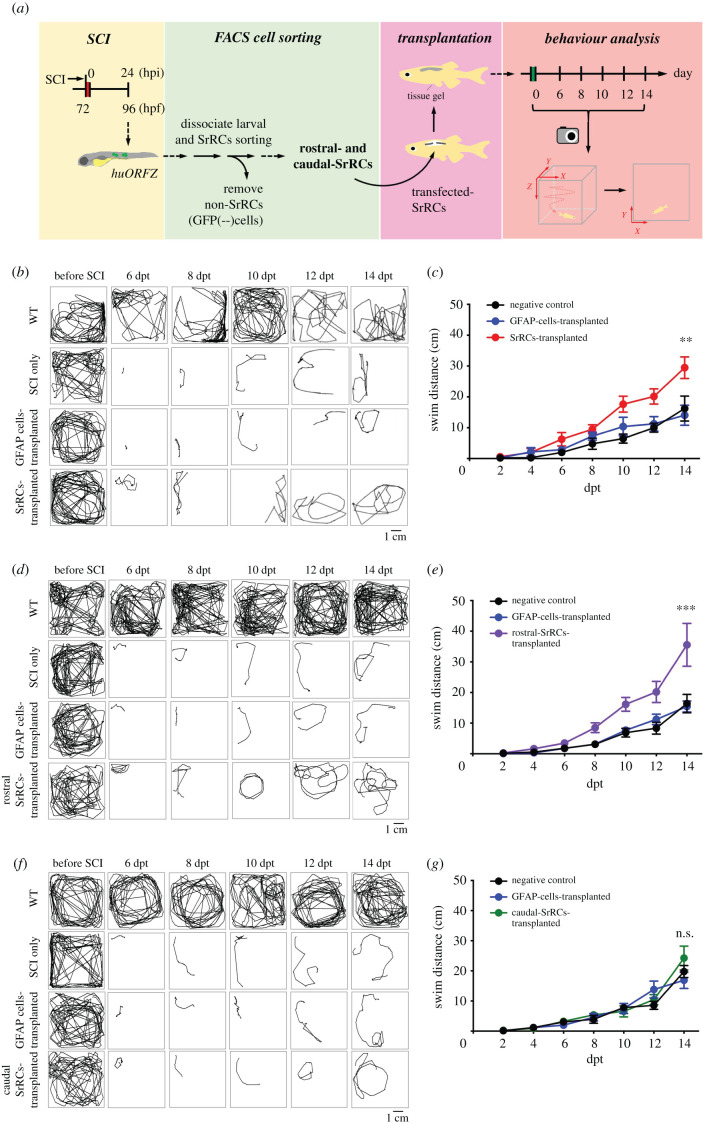
Unlike transplanted caudal-SrRCs, transplantation of rostral-SrRCs could significantly improve neuronal regeneration of SCI-adult zebrafish. (*a*) Schematic diagram illustrating the effect of SrRCs transplantation on neuronal regeneration of SCI-adult fish. SCI was performed on *huORFZ* embryos at 72 hpf (hours-post-fertilization). (*b–g*) The locomotive route of each fish was tracked in line, and its swimming distance (cm) was calculated. The results obtained from non-treated WT (WT; positive control), SCI-treated zebrafish (SCI-only; negative control) and GFAP-cell transplantation (sham control) were compared to (*b*,*c*) SrRCs transplantation, (*d*,*e*) rostral-SrRCs transplantation and (*f*,*g*) caudal-SrRCs transplantation. Two-way ANOVA with Bonferroni multiple comparisons test (***p < 0.01, **p < 0.1; *n* = 10). Error bars indicate s.e.m. dpt: days post-transplantation; ns: not significant.

To doubly confirm that rostral-SrRCs played a major role in neuronal regeneration after SCI, we performed cell transplantation *in vivo,* in which SrRCs sorted from SCI-larvae were transplanted into SCI-adult zebrafish, followed by an analysis of their swimming performance. The entire flow chart of this experiment is illustrated in figure 5*a*. Seven experimental groups were designed as described in Material and Methods. The touch-evoked response approach demonstrated that the swimming distance of the SrRCs-transplanted adults was 2.5-fold longer compared to that of the SCI-adults during the 10-day recovery period (figure 5*b*,*c* and electronic supplementary material, movie S5), suggesting that SrRCs transplantation could improve neuronal regeneration during the recovery of SCI-adult zebrafish, as supported by both light-stimulated and touch-evoked response. Since these two approaches produced essentially equivalent results, as noted above, we used the touch-evoked response for further study. When we transplanted rostral-SrRCs alone, the swimming distance of these adults was 3.29- and 3.5-fold longer compared to the RG-cell-transplanted SCI-adults after recovering for 10 and 14 days, respectively (figure 5*d*,*e*), while swimming distance showed no significant difference between the adults transplanted with caudal-SrRCs alone and that of RG-cell-transplanted SCI-adults (figure 5*f*,*g*), suggesting that rostral-SrRCs do play a major role in the axonal regeneration of SCI-adult zebrafish compared to caudal-SrRCs.

### 2.10. Using transcriptomic analysis to screen key regulatory genes involved in neuronal regeneration after spinal cord injury

**Figure 6.**
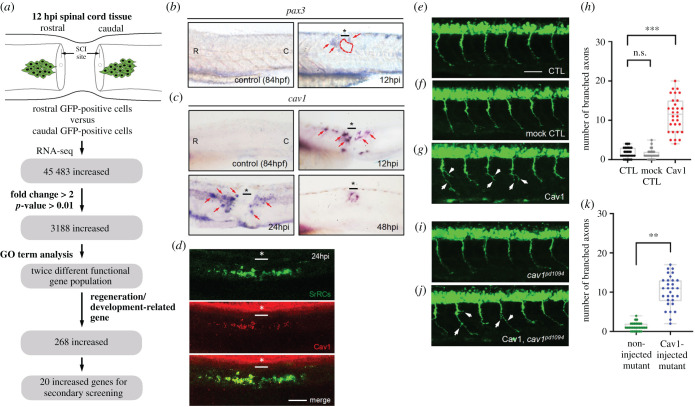
Expression of *cav1* transcript and encoded protein in zebrafish embryos. (*a*) Schematic illustration showing how putative genes were selected for this study. Using whole-mount *in situ* hybridization to detect (*b*) *pax3* and (*c*) *cav1* mRNAs in the spinal cord of zebrafish embryos before SCI (served as a negative control) and after SCI at 12, 24 and 48 h post-injury (hpi), as indicated. Since the expression pattern of *pax3a*, another transcript screened from RNA-seq, also showed increased expression in rostral-SrRCs compared to that in caudal-SrRCs, it served as a positive control in a parallel experiment. (*d*) Using IHC detection to examine the protein level of Cav1 (labelled by red signal) expressed in SrRCs (labelled by green signal), including rostral-SrRCs and caudal-SrRCs, in SCI-embryos at 24 hpi. The underscore stars indicate the SCI sites. Scale bar shown at lower right corner is 50 μm. (*e*) CaP motor neuron labelled by GFP was observed in the spinal cord of zebrafish embryos from transgenic line *Tg(mnx:GFP)* at 24 hpf (un-injected control. Student's *t*-test was used to perform statistical analysis, **p < 0.01 significance. CTL), (*f*) pCS2-vector-injected embryos (mock control) and (*g*) pCS2-Cav1-injected embryos. (*h*) Quantification and comparison of the number of branched axons of CaP motor neurons between groups. (*i*) GFP-labelled CaP motor neurons observed in the spinal cord of embryos from *cav1* mutant (*cav1^pd1094^*) at 24 hpf (un-injected control). Student's *t*-test was used to perform statistical analysis, **p < 0.01 significance. (*j*) pCS2-Cav1-injected embryos from a double-transgenic line, in which mutant *cav1^pd1094^* was crossed with *Tg(mnx:GFP). *White arrows indicate branched axons.** (*k*) Quantification and comparison of the number of branched axons of CaP motor neurons between groups as indicated. Each spot indicates the number of axonal branches in each embryo (*n* = 30), in which six CaP motor neurons were analysed. Student's *t*-test was used to perform statistical analysis, ***p < 0.001 significance.

To determine genes in rostral-SrRCs that play major roles in neuronal regeneration, we separately collected rostral- and caudal-SrRCs from SCI-embryos at 12 hpi, followed by RNA sequencing analysis (figure 6*a*). A total of 11 359 transcripts were increased in rostral-SrRCs, of which 3188 mRNAs were significantly upregulated (Fold change greater than 2, *p*-value > 0.01) (electronic supplementary material, figure S8A,B). Based on Gene Ontology analysis, most of these genes were related to regeneration and development (ex: CNS development and cell differentiation) (electronic supplementary material, figure S8C,D). On the other hand, a total of 12 839 transcripts were increased in caudal-SrRCs, of which 3911 mRNAs were significantly upregulated (Fold change greater than 2, *p*-value > 0.01). Most of these genes were related to biological processes (e.g. oxidation-reduction and metabolic pathway). At the first step, we focused on the genes significantly elevated in rostral-SrRCs. Specifically, we focused on *Caveolin-1 (cav1)* transcripts which were increasingly expressed in the rostral site after SCI at 24 and 48 hpi compared with its expression in caudal-SrRCs (figure 6*b*,*c*). Immunohistochemical analysis was then used to detect SCI-embryos. Compared to caudal-SrRCs, we also found that Cav1 protein was predominantly expressed in rostral-SrRCs (figure 6*d*). Additionally, Cav1 controls cell proliferation and cell death by suppressing the expression of Survivin, an inhibitor of apoptosis protein. Zebrafish Cav1 is an essential factor for heart and liver regeneration [32,33]. Therefore, in this study, we hypothesized that Cav1 might also play a critical pro-regenerative role after SCI in zebrafish.

### 2.11. Overexpression of Cav1 in zebrafish embryos induced more active axonogenesis *in vivo*

To examine whether Cav1 plays a role in motor neurons, we employed zebrafish transgenic line *Tg(mnx:GFP),* in which the caudal primary motor neuron (CaP) was labelled by GFP. Plasmid construct pCS2-Cav1 was then injected into *Tg(mnx:GFP)* embryos. Compared to the un-injected control group and pCS2-vector-injected mock group, a significant increase in the number of branched axons of CaP motor neurons was observed (figure 6*e*,*h*). We further employed heterozygous embryos derived from a double-transgenic line, in which *Tg(mnx:GFP)* was crossed with a *cav1* mutant line, *cav1^pd1094^*. As shown in figure 6*i*, pioneering motor axon guidance defects were observed in *cav1* mutant embryos. However, when pCS2-Cav1 was injected into these *cav1* mutant embryos, the defect was rescued **(**figure 6*j*), and we saw a significant increase in the number of CaP motor branch axons (figure 6*k*). Collectively, it can be concluded that Cav1 induces axonal outgrowth in CaP motor neurons at an early developmental stage.

Additionally, we performed an experiment in which *cav1-flag* mRNA was microinjected in the embryos derived from double-transgenic line *Tg*(*mnx:GFP*) crossed with *cav1^pd1069^* to overexpress Cav1-Flag fusion protein. We found that positive signals against Cav1-Flag were observed in the axon and axon branching-promoting sites using immunoblotting with antibody against Flag reporter (electronic supplementary material, figure S9), suggesting Cav1 can express in the axon and axon branching-promoting sites to promote axonal outgrowth.

### 2.12. Caudal-SrRCs transfected with *cav1* caused significant enhancement of neurite outgrowth *ex vivo*

**Figure 7.**
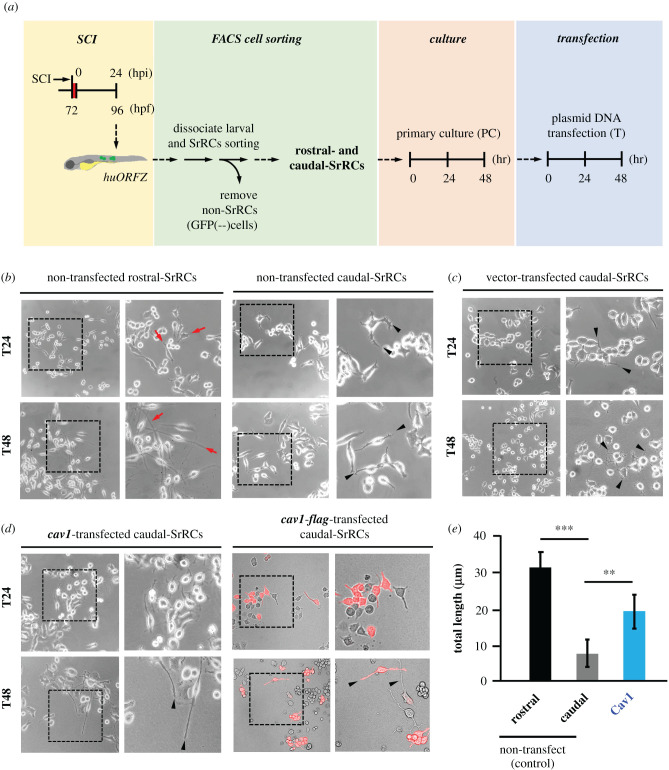
Overexpression of *cav1* could drive caudal-SrRCs to extend longer axonal neurites *in vivo*. Rostral- and caudal-SrRCs were separately collected from SCI-embryos, and primary culture (PC) was carried out for 48 h. Afterwards, plasmid containing *cav1* cDNA was transfected into caudal-SrRCs. Neurites developed from cultured SrRCs after plasmid was transfected for 24 h (T24) and 48 h (T48) were morphologically examined: (*a*) non-transfected rostral-SrRCs, (*b*) non-transfected caudal-SrRCs, (*c*) caudal-SrRCs transfected with pCS2-vector and (*d*) caudal-SrRCs transfected with pCS2 containing *cav1* and *cav1-flag* cDNA. Right panel of each figure shows the amplified image of the box area indicated on left panel. Arrowheads indicate the extended axons. (*e*) The average neurite length developed from SrRCs cultured for 48 h after transfection was calculated from 200 cultured SrRCs. Statistical analysis was based on *t*-test at **p < 0.01 and ***p < 0.001 significance.

To support the hypothesis that reduced expression of *cav1* in caudal-SrRCs might downregulate the capacity of caudal-SrRCs to participate in neuronal regeneration, we isolated the caudal-SrRCs, transfected with pCS2-vector containing *cav1* and then performed primary culture (figure 7*a*,*b*). Compared with caudal-SrRCs transfected pCS2-vector only (served as control group) (figure 7*c*), *cav1*-transfected caudal-SrRCs (*cav1*-caudal-SrRCs) displayed a 2.52-fold improvement in neurite outgrowth (figure 7*d*,*e*) after transfection for 48 h (T48) on the basis of a 60% transfection rate (electronic supplementary material, figure S10). Using Anti-Flag antibody, we also clearly demonstrated that recombinant Cav1 was present in caudal-SrRCs transfected with pCS2-Cav1-Flag at T24 and T48 with (figure 7*d*; red signal). Therefore, Cav1 appears to play a dominant role in the enhancement of neurite outgrowth during nerve repair *ex vivo*. Since *cav1* is more predominantly expressed in rostral-SrRCs compared to caudal-SrRCs, we suggest that this line of evidence further supports rostral-SrRCs as a potential major player in neuronal regeneration after SCI.

### 2.13. Transplantation of Cav1-caudal-SrRCs to SCI-adult zebrafish could improve recovery of swimming ability

**Figure 8.**
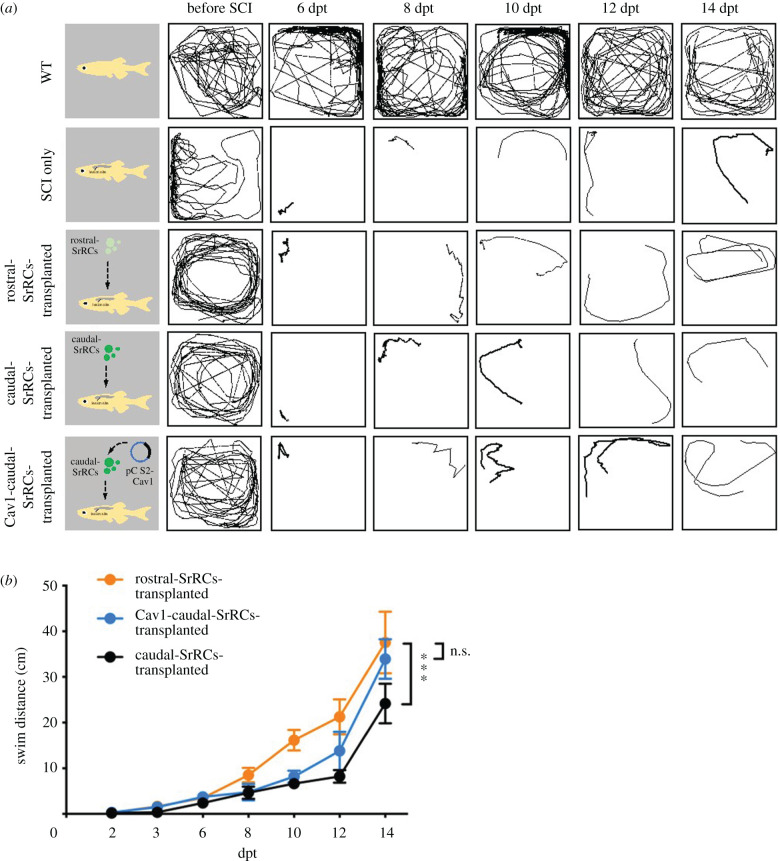
Transplantation of Cav1-transfected caudal-SrRCs improved neuronal regeneration of adult zebrafish post-SCI. Locomotion test of SCI-adult fish during neuronal regeneration from 6 days post-transplantation (dpt) to 14 dpt. (*a*) The locomotive route of each fish was tracked in line, (*b*) its swimming distance (cm) was calculated, and the results were compared among non-treated WT-adult (WT; positive control), SCI-adult (SCI-only; negative control), rostral-SrRCs-transplanted SCI-adult, caudal-SrRCs-transplanted SCI-adult and Cav1-caudal-SrRCs-transplanted SCI-adult in which SCI adults were transplanted with caudal-SrRCs transfected by *cav1*. Two-way ANOVA and *t*-test were used to perform statistical analysis with different significance level at ***p < 0.001, **p < 0.05, *p < 0.01; error bars indicate s.e.m.

To further confirm this difference of effect on neuronal regeneration between rostral- and caudal-SrRCs *in vivo*, we collected rostral- and caudal-SrRCs separately and performed primary cell culture. After culturing cells, we transplanted either rostral- or caudal-SrRCs to SCI-adult zebrafish at the lesion site and evaluated swimming behaviour analysis. According to the results shown in figure 8*a*,*b* and electronic supplementary material, movie S6, swimming behaviour analysis at 48 h after transplantation demonstrated that (i) the swimming distance of rostral-SrRCs-transplanted SCI-adults exhibited a 1.79-fold increase over that of caudal-SrRCs-transplanted SCI-adults, suggesting that rostral-SrRCs have a greater effect on neuronal regeneration for SCI-adult zebrafish compared to caudal-SrRCs; (ii) the swimming distance of *cav1*-caudal-SrRCs-transplanted SCI-adults exhibited a 1.32-fold increase compared to caudal-SrRCs-transplanted SCI-fish, suggesting that Cav1 can improve the nerve repair capacity of caudal-SrRCs; and (iii) the swimming distance of *cav1*-caudal-SrRCs-transplanted SCI-fish was close to that of rostral-SrRCs-transplanted SCI-adults, suggesting that addition of *cav1* expression could enhance the NDC of caudal-SrRCs, improving neuronal recovery. Based on such *ex vivo* results, we conclude that the neuronal regeneration capability of caudal-SrRCs can be improved by the overexpression of Cav1 in cells.

### 2.14. Overexpression of zebrafish Cav1 resulted in much more extended axonal neurites in mammalian cells and increased expression of neuronal genes

**Figure 9.**
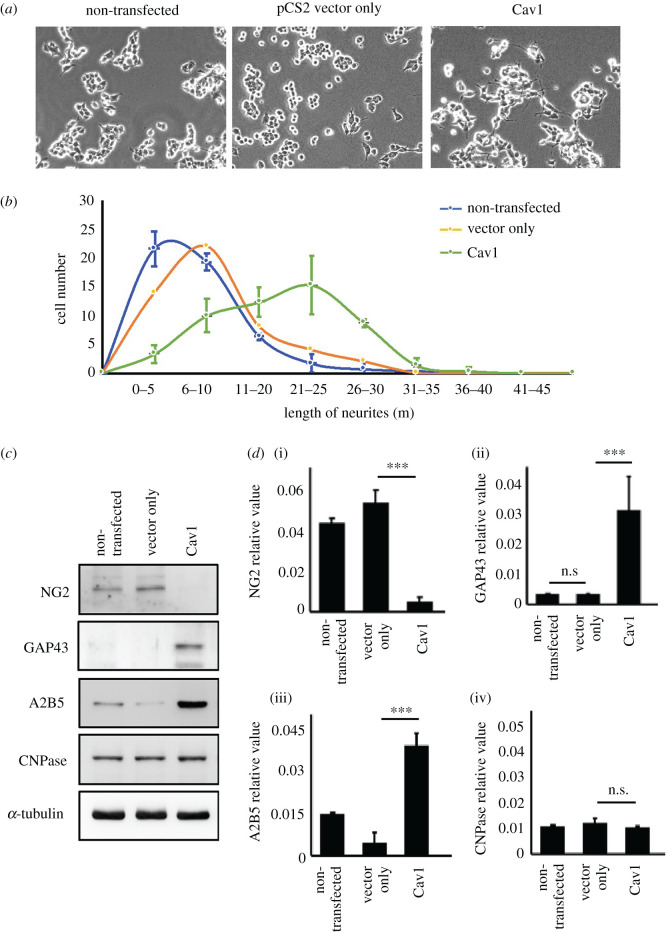
Overexpression of *cav1* in motor neuron cells can promote neurite outgrowth and expression of functional neuron markers. NSC34 motor neuron cells were employed to examine the effect of Cav1 on neurite outgrowth. NSC34 cells were grouped into three treatments: non-transfected (control group), transfected with pCS2-vector (pCS2-vector only group) and transfected with pCS containing *cav1* cDNA (pCS2-*cav1* group). (*a*) Neurite outgrowth of motoneurons developed from 48 h-cultured NSC34 cells overexpressing *cav1* (pCS2-*cav1* group) was observed under microscopy. (*b*) The size distribution of neurite length developed from NSC34 cells transfected with materials as indicated. Cell number with various lengths of neurites after culturing for 48 h was determined (50 cells per experimental condition). (*c*) Western blot analysis using antibody against neuron markers as indicated. Three independent experiments were performed. The α-tubulin served internal loading control. (*d*) Relative expression level of each marker was quantified. Statistical analysis used *t*-test with different significance levels at ***p < 0.001, **p < 0.05, *p < 0.01; error bars indicate s.e.m. n.s.: not significant.

We asked if the expression of zebrafish Cav1 in mammalian neuronal cells would promote neuronal development. To address this question, we employed mammalian motoneuron NSC34 cells overexpressing Cav1. The average length of neurite outgrowth developed from Cav1-transfected NSC34 cells exhibited 1.93-fold longer axonal outgrowth than that of pCS2-vector-transfected NSC34 cells (figure 9*a*,*b*). The number of cells displaying longer neurite length (greater than 20 um) in the Cav1-transfected NSC34 cells significantly exceeded that of the control group (figure 9*b*), suggesting that Cav1 expression could promote longer axonal development in more mammalian nerve cells.

Next, we further demonstrated that Cav1-mediated axonal outgrowth results from proteins involved in neuronal differentiation, as induced by Cav1 overexpression in neuronal cells. When Cav1 was transfected into NSC34 cells, the levels of such endogenous proteins as NG2 (restricts axonal regeneration and inhibits neurite outgrowth), GAP43 (axonal regeneration), A2B5 (astrocyte/glial progenitor) and CNPase (oligodendrocyte and Schwann cell marker) were studied. Compared with two negative controls (non-transfected cells and pCS2-vector-transfected cells), endogenous NG2 was significantly deceased in Cav1-transfected NSC34 cells, while GAP43 and A2B5 were increased. Meanwhile, expression of the oligodendrocyte and Schwann cell marker CNPase remained unchanged (figure 9*c*,*d*), most likely because NSC34 is a motoneuron-like cell without containing oligodendrocytes and Schwann cells, meaning, in turn, that oligodendrocyte and Schwann cell marker CNPase was not changed in Cav1-overexpressing NSC34. Therefore, we concluded that overexpression of zebrafish Cav1 can promote axonal development in mammalian neurons through decreasing NG2, but increasing GAP43 and A2B5.

## 3. Discussion

**Figure 10.**
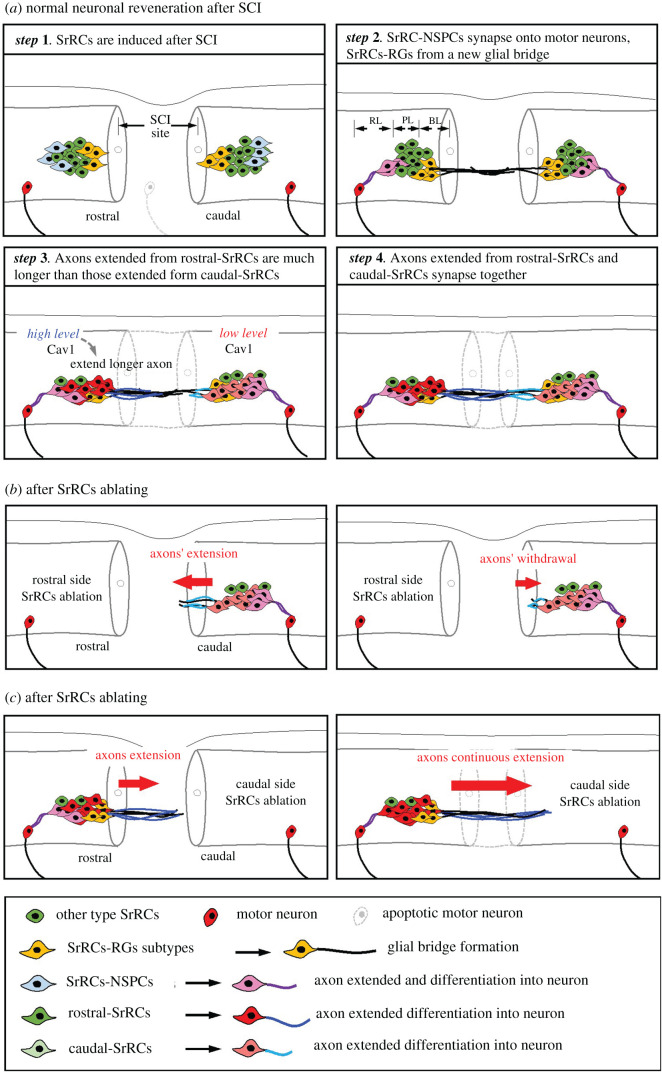
Schematic illustration showing the contribution of SrRC subtypes to neuronal regeneration after SCI in zebrafish. (*a*) Several steps of normal neuronal regeneration. (*b*) Ablation of rostral-SrRCs attenuates the elongation of caudal-SrRCs axons, effectively aborting neuronal repair. (*c*) Ablation of caudal-SrRCs has little effect on axons derived from rostral-SrRCs, which continue to develop and perform neuronal repair.

In this work, we asked whether any specific cell population is involved in neuronal regeneration after SCI. Results showed that a specific cell population, termed SrRCs, is highly responsive to laterally mechanical SCI stress and actively participates in neuronal regeneration (figure 10*a*). SrRCs are mostly composed of two main cell types, RGs and NSPCs, but also a few OLS and OLPs (figure 10*a*, Step 1). More importantly, we found subtype proportionality among SrRCs responsive to SCI stress in zebrafish. Specifically, after mechanical SCI in zebrafish embryos, the NSPCs-SrRCs subtype develops axons to promote connection with undamaged motor neurons, while the RGs-SrRCs subtype forms glial bridges (figure 10*a*, Step 2). Other subtypes of SrRCs differentiate into neurons and extend axons to connect with each other and surviving motor neurons, resulting in neuronal regeneration across the lesion. Interestingly, we found that the subset of subtype cells that form rostral-SrRCs can elongate their axons to a greater degree compared to that subset of subtype cells which forms caudal-SrRCs (figure 10*a*, Step 3–4). Ablation of rostral-SrRCs resulted in the withdrawal of axons initially developed from caudal-SrRCs (figure 10*b*), while ablation of caudal-SrRCs had little effect on axons developed from rostral-SrRCs, which were able to continuously develop (figure 10*c*). Therefore, although SrRCs are present on both sides of SCI lesion, we propose that the NDC of rostral-SrRCs is far higher than that of caudal-SrRCs, resulting in the greater functionality of rostral-SrRCs with respect to axonal regeneration after SCI in zebrafish. This hypothesis was strongly supported by our cell transplantation experiment, which demonstrated that the swimming performance of SCI-adults transplanted with rostral-SrRCs exceeded that of SCI-adults transplanted with caudal-SrRCs during recovery.

After performing SCI on zebrafish, different cell populations have been reported to participate in neuronal repair [34]. Using markers, different cell subtypes were identified, such as SOX2(+)/GFAP(+), A2B5(+)/GFAP(–) and A2B5(+)/GFAP(+), as well as GFAP(+)/BrdU(+), GFAP(+)/BrdU(−) and GFAP(–)/BrdU(+) [14,16], suggesting that many subtypes exist in a single cell type. However, these studies did not settle the questions of constituents and proportionality among subtypes in different cell populations actively engaged in neuronal regeneration encountered at different stresses. Here, we addressed this question and found a stress-resistant subtype cell population, SrRCs, easily identifiable through the expression of GFP in *huORFZ* embryos, a transgenic zebrafish that is very responsive to ER stress [34]. These SrRCs consist of 32% RGs, 16% NSPCs, 10% OLPs and 4% OLs, including 3.4% SOX2-GFAP-mixed cells. Nevertheless, Zeng *et al*. [21] exposed *huORFZ* embryos to hypoxic stress and also found a subtype cell population, termed hypoxia-responsive recovering cells (HrRCs), in the spinal cord. These HrRCs consist of 36% NSPCs, 26% RGs, 13% OLPs and 3% OLs, including 5.2% SOX2-GFAP-mixed cells [21]. This evidence suggests that different stresses activate different cell populations, finally comprising a complex of subtypes in different proportions to actively engage in neuronal regeneration at the site of the lesion. For example, the major subtype involved in neuronal regeneration of SCI-treated embryos is RGs, while in hypoxia-exposed embryos, it is NSPCs. Importantly, in both cases, these GFP-expressing subtype cell populations are highly responsive and alive under stress microenvironments, and they were shown to play a key role in neuronal regeneration in the spinal cord of zebrafish.

RGs are well known as an important cell type involved in neuronal regeneration. Among the specific subtype-cell population of SrRCs found in this study, we discovered that the RGs-SrRCs subtype plays a key role in neuronal repair after SCI. Specifically, the RGs-SrRC subtype contributes to the formation of new glial bridges at the injury site, while the RGs-non-SrRC subtype does not. Therefore, we have identified the RGs-SrRCs subtype as stress-resistant and able to play a predominant role in neuronal regeneration following mechanical SCI lesion. This complicated cell heterogeneity also exists in the spinal cord of zebrafish after hypoxic stress [21]. Assinck *et al*. [35] and Vismara *et al*. [36] reported that transplantation of a single cell type may promote effective recovery for therapy following SCI. However, based on these studies, the transplantation of stress-resistant cells in the proper proportion for each cell subtype, depending on the stressor, is recommended for effective neuronal repair therapy.

Among the cell population of SrRCs, the RGs subtype is induced at a higher comparative proportion following mechanical SCI lesion. This finding is congruent with that of Mokalled *et al*. [17], who reported that glial bridge formation can promote neuronal regeneration. Thus, we speculate that the RGs-SrRCs subtype is required for glial bridge formation during the early stage of neuronal regeneration. Meanwhile, OLPs and OLs are also included among other cell subtypes belonging to SrRCs, but in a relatively lower proportion. Ohnmacht *et al*. [28] reported that OLPs are able to proliferate, differentiate and develop into mature OLs, which then form myelinated axons after spinal cord lesion. Therefore, we speculate that OLPs-SrRCs may also contribute to neuronal regeneration by forming myelin during neuronal recovery.

Focusing on glial bridge formation after SCI, many reports have concluded that some neurons have a regenerative ability, while others undergo apoptosis following injury [16,17,37,38]. Such apoptosis explains the loss of connection with undamaged neurons nearby the lesion site at an early stage. During regeneration, newly formed axons must connect with undamaged neurons neighbouring the lesion site. However, the precise cell subtypes involved in regenerative connection with undamaged neurons to promote functional locomotor recovery after SCI remains to be elucidated. In mammals, axons of neurons in the spared, but reactive, neuronal tissue (SRNT) located at both sides of the non-lesion site have shown potential growth responses [39]. After SCI, axons in the SRNT region functionally synapse with newly formed neuronal axons, if any [40,41]. Corresponding to mammalian SRNTs, a PL area exists in zebrafish, and it contains many proliferating cells actively differentiating into neurons [15]. However, we found that only the NSPC-SrRCs subtype could elongate axons and connect with undamaged motor neurons during the neuronal repair. This line of evidence suggests that the zebrafish PL area contains an abundance of SrRCs subtypes that contribute to neuronal repair, whereas mammalian SRNTs do not contain such an abundance of subtype cells. These contrasting facts provide a clue that might explain why the functional recovery of spinal cord locomotion is limited in mammals after SCI.

Mokalled *et al*. [17] reported that *ctgfa* mRNA is expressed in RGs and that its encoded CTGF is necessary to stimulate glial bridging and natural spinal cord regeneration after injury [17]. We found *ctgfa* mRNA to be expressed in both rostral- and caudal-SrRCs after SCI [17], consistent with the results described by Mokalled *et al*. [17]. Nevertheless, we found a predominance of *ctgfa* mRNA and encoded CTGF expressed in rostral-SrRCs compared to caudal-SrRCs, providing still more evidence that rostral-SrRCs play a functionally key role in neuronal regeneration, thus strengthening our conclusions from the above experiments. We also note that mammalian CTGF contributes to glial scar formation after CNS lesion [42], whereas zebrafish CTGF promotes glial bridge formation. We found that *ctgfa* mRNA and encoded CTGF reached their highest expressions at 36 hpi, corresponding to the time of glial bridge formation in zebrafish [17], as described by Mokalled *et al*. [17]. Expressions of *ctgfa* mRNA and CTGF became less evident at 48 hpi.

Cav1 protein is an essential factor in zebrafish heart regeneration [33] and liver regeneration [43]. Interestingly, in this study, we found that Cav1 is also involved in neuronal regeneration of spinal cord. The *cav1* mRNA was highly expressed around the SCI site. Specifically, based on RNA-seq, the expression of *cav1* was more predominant in rostral-SrRCs than in caudal-SrRCs. In fact, rostral-SrRCs exhibit a higher regenerative capacity because they contain more *cav1*-expressing cells, which, in turn, direct a predominance of rostral-SrRCs to the lesion site after SCI. We also demonstrated that caudal-SrRCs harbouring *cav1* can improve their NDC, thereby potentiating, to some degree, normal recovery and neuronal regeneration. Axonogenesis is critical for maintaining the polarized structure of neurons [44]. More specifically, axonogenesis governs the generation and outgrowth of axons during neuronal development [45] and regeneration [46]. In detail, we see that axonogenesis was inhibited in *insm1a*-knockout *Tg(mnx:GFP)* embryos, resulting in impeding retinal regeneration owing to CaP pioneering motor axon guidance defects [47]. Similarly, in the present study, we employed heterozygous embryos derived from a double-transgenic line, in which *Tg(mnx:GFP)* was crossed with a *cav1* mutant line, *cav1^pd1094^*, and pioneering motor axon guidance defects were observed in *cav1* mutant embryos. However, when pCS2-Cav1 was injected into these *cav1* mutant embryos, the defect was rescued**,** and we saw a significant increase in the number of CaP motor branch axons.

We also note that Cav1 is required not only for glucose-induced CTGF upregulation in the mesangial cells of the kidney [48], but also for high CTGF expression in hepatocytes [49]. Pavlides *et al*. [50] demonstrated that the loss of Cav-1 in stromal cells induces ligand-independent activation of the TGF*β* pathway, resulting in the increased transcription of the TGF*β* target gene CTGF [50,51]. This line of evidence demonstrates that the presence of Cav1 can impact CTGF expression in the stromal cells, kidney mesangial cells and hepatocytes. In this study, we reveal that high expression of Cav1 and CTGF in rostral-SrRCs plays a key role in neuronal regeneration since rostral-SrRCs promote axonal growth, axon branching and swimming ability after SCI. Specifically, the RGs-SrRCs subtype can express Cav1, contributing to the formation of glial bridges. Meanwhile, Mokalled *et al*. [17] reported that *ctgf* transcripts are highly expressed in the rostral side of SCI site compared to their expression in the caudal side of SCI-embryos. Furthermore, they showed that CTGF is necessary to stimulate glial bridging and natural spinal cord regeneration after injury [17]. Taken together, it is reasonable to speculate the likelihood that Cav1 and CTGF may share the same signalling pathway involved in neuronal regeneration after SCI. Overall, our findings present a novel mechanism for the regulation of Cav1 signalling, which is critical for neuronal regeneration. More detailed studies are needed in the future to investigate the molecular mechanism controlling the interaction between Cav1 and CTGF in the SCI repair process.

In sum, we learned that upregulation of Cav1 promotes (i) axonogenesis *in vivo* (figure 6) and *in vitro* (figure 9); (ii) axon formation and elongation *ex vivo* (figure 7) and (iii) neuronal regeneration and functional recovery using SrRCs transplantation to SCI-adult zebrafish (figure 8). Additionally, we demonstrated that *cav1* mRNA is predominantly present in rostral-SrRCs at the early regeneration stage of 12 hpi, reaching its highest level at 24 hpi and then starting to decline at 48 hpi (figure 6*a*,*c*). Meanwhile, rostral-SrRCs start to extend axons at 36 hpi, connecting to caudal-SrRC axons at 48 hpi (figure 2*c*). It appears that the ability of rostral-SrRCs to process axonogenesis and regeneration at late regeneration stage (after 24 hpi) is dependent on the presence of Cav1 at high levels during the early regeneration stage (12 to 24 hpi). Therefore, we can conclude that Cav1 is a critical protein for axonogenesis and regeneration after SCI in zebrafish. However, we noticed that the improved locomotion recovery for SCI-zebrafish, as driven by the transplanted caudal-SrRCs harbouring *cav1*, cannot match that of SCI-zebrafish transplanted by rostral-SrRCs. This finding shows that *cav1* might cooperate with other gene(s) in a manner that causes rostral-SrRCs to play a more comprehensive role in neuronal regeneration than previously thought. It is also reported that Cav-1 plays a critical role in suppressing ER stress-induced cell apoptosis through the activation of p38 mitrogen-activated protein kinase pro-survival pathway [52]. Additionally, Cav1 not only enhances cell migration in a number of cell types, but it is also essential for neurite outgrowth in neuronal types dependent on tyrosine-14 phosphorylation by Src family kinases [53]. Additional work will be necessary to determine whether tyrosine-14 phosphorylation and/or Src family kinases impact Cav1 to contribute to neurite outgrowth and migration of SrRCs during the neuronal regenerative process.

Taken together, the above findings support the hypothesis that rostral- and caudal-SrRC are involved in neuronal regeneration of SCI-treated zebrafish, but that rostral-SrRCs play a more prominent role because rostral-SrRCs exhibit a higher expression of factors such as CTCF and Cav1. Nevertheless, future studies must be performed to decisively understand the unique biological characteristics of rostral-SrRCs.

## 4. Material and methods

### 4.1. Zebrafish

Zebrafish WT strain AB/TU and transgenic lines, including Tg(*GFAP:dTomato*) [54], Tg(*huc:dsred*) [55], *huORFZ* [27] and *cav1^pd1094^* mutant [33], were cultured. Embryo medium was replaced by fresh medium containing 0.003% 1-phenyl-2-thiourea at 24 hpf to reduce pigmentation.

### 4.2. Mechanical crush injury to induce spinal cord injury of zebrafish larvae and adults

The 72-hpf *huORFZ* embryos were immersed in embryo medium containing 0.02% tricaine for anaesthesia. A fine glass needle with a 0.15 mm opening was employed to perform spinal cord crush lesion. After treatment, these embryos were placed in phosphate-buffered saline (PBS) for 24 h. To prepare mechanical SCI for adults, we followed the procedures described by Zeng *et al*. [56].

### 4.3. Confocal microscopy and image processing

Fluorescence signals were captured by a Zeiss confocal microscope (LSM 780) and Nikon confocal laser-scanning microscope (A1R). Images were analysed using ImageJ and ZEN2009 Light Edition. For time-lapse imaging of embryos (figures 2 and 4), we embedded embryos into a 2–3% methylcellulose gel for image acquisition and released to culture media every 3 (or 6) h.

### 4.4. Whole mount *in situ* hybridization

The procedures were described by Zeng *et al*. [21]. Images were captured using a light stereomicroscope with a CCD camera (MZ FLIII, Leica).

### 4.5. Microinjection and mRNA synthesis

The procedures of microinjection and mRNA synthesis were described by Lee *et al*. [27] except that plasmid pCS2-Cav1-Flag was linearized by NotI. The capped *cav1-flag* mRNA was generated by the SP6 Message Machine Kit (Ambion). It was diluted to working concentration of 44 ng µl^−1^ prior to each 2.3 nl injection.

### 4.6. Observation of motor axon phenotype

We followed the procedures described by Bremer and Granato [57], except that embryos were injected with linear form plasmid DNA at a concentration of 30 ng. At 24 hpf, embryos were mounted with 4% methyl cellulose, and a z stack was imaged using a Zeiss confocal microscope (LSM 780, Carl Zeiss AG). The number of CaP motor branch axons was then analysed using image processing software combined with a Zeiss LSM 780 (Carl Zeiss AG). In total, 30 embryos were studied in each group, and six axons within three somites in the trunk of each embryo were analysed.

### 4.7. Dissociation and immunostaining of embryonic cells

The procedures for dissociation were described by Lee *et al.* [27], and for immunostaining, the procedures were described by Zeng *et al*. [21].

### 4.8. Fluorescence-activated cell sorting

A FACSAria cell sorting system (BD Biosciences, FACSVerse™) and SH800 cell sorter (SONY) were used to perform FACS to sort GFP(+) cells under sterilized condition according to the protocols described by Dobson *et al*. [58] and Vitak *et al*. [59].

### 4.9. Western blot analysis

Total proteins extracted from embryos were analysed on a 10% SDS-PAGE followed by Western blot analysis according to the procedures described by Zeng *et al.* [21], except that the antibodies against NG2 (N8912; 1:100), Growth Associated Protein 43 (GAP43) (RRID:AB_443303; 1:1000), A2B5 (AB53521; 1:500), CNPase (ab227218; 1:1000), α-tubulin (RRID:AB_477579; 1:5000), goat anti-mouse-HRP (RRID:AB_955439; 1:5000) and goat anti-rabbit-HRP (RRID:AB_631746; 1:5000) were used.

### 4.10. Primary culture

Cells from the rostral and caudal sides of SCI-embryos were separately dissociated at 24, 36 and 48 hpi by following the procedures described by Ho *et al*. [60] and Zeng *et al*. [21].

### 4.11. *In vivo* cell ablation

The optical system of the infrared laser-evoked gene operator (IR-LEGO) microscope (Japan) described in Kamei *et al*. [61] was employed to perform cell ablation by following the protocol described by Kimura *et al.* [62] and Zeng *et al*. [21] with some modifications. Briefly, larvae were embedded in 4% methylcellulose for stable positioning of the targeted cells. The targeted GFP(+) cells at rostral, caudal and both sides of the SCI site were ablated with a high-power flash irradiation of 75 mW IR laser (1480 nm) for 8 ms. After the IR laser had been used, the treated embryos were studied at the starting time (IR0), 12 h post-irradiation (IR12), IR24, IR36, IR48 and IR72, individually.

#### 4.11.1. Location and quantification of the number of NSPCs- and RGs-SrRCs

The immunofluorescence signals from rostral and caudal sides of the lesion site were quantified on captured images of whole-mount samples. Samples were imaged using a compound fluorescence confocal microscope (Zeiss LSM 780). Based on the definition proposed by Briona *et al*. [15], images of the rostral side of the SCI site were divided into (i) the BL area within 30 µm of the SCI site, (ii) the PL area within 30 µm of rostrum and (iii) the RL area adjacent to PL within 60 μm of rostrum. We quantified the pixel area of RFP/GFP-double signals by ImageJ software (*n* = 10).

### 4.12. Locomotive activity analysis

#### 4.12.1. Touch-evoked response

Using microscopy combined with a camera system (Leica M205FA), we observed the touch-evoked response of individual larvae every 12 h after irradiation. Four groups were designed: (i) positive control receiving neither SCI nor IR-laser, (ii) negative control receiving SCI without ablating GFP(+) cells, (iii) sham control receiving SCI with ablation of GFP(−) cells and (iv) ablation group receiving SCI with ablation of GFP(+) cells. At IR72, larvae were gently touched at the top of the head with a blunt tip. The movement of every larva was recorded for 15 s by video under the condition of exposure time for 60 ms, gain at 3, zoom at 0.781 and intensity at 0.

#### 4.12.2. Locomotion assay

At IR72, six larvae were randomly selected from each group. Each larva was kept in one well of a 24-well plate. When their heads were touched, a high-speed camera was used to record their locomotion, and UMATracker was used to quantify swimming distance and route of movement. After cell transplantation, swimming performance was evaluated on the basis of swimming distance of adult zebrafish within a fixed time. Five groups were designed: (i) non-treated WT adults as positive controls; (ii) SCI-treated adults as negative controls; (iii) transplantation of GFAP cells sorted from Tg(*gfap*:dsRed) embryos at 96 hpf as sham controls; (iv) transplantation of rostral-SrRCs sorted from SCI-*huORFZ* embryos; and (v) transplantation of caudal-SrRCs.

#### 4.12.3. Swimming distance assay

A high-speed camera (Photron, FASTCAM SA1.1) was used to record larval swimming performance *in vivo.* Each individual larva was recorded three times, and UMATracker was used to analyse the larval swimming route. The route sampled from the video was converted into a coordinate. Then, each coordinate was converted into swimming distance in mm by Excel. Data were presented as an average from 18 larvae. Quantification of locomotion in adult zebrafish was performed using Dipp-AAM software (Ditect, Japan), as previously described by Shimmura *et al.* [63]. Each adult was recorded three times at each examined time point. Data were presented as an average from ten samples (*n* = 10).

### 4.13. Cell transplantation

GFP(+) cells were sorted out from the SCI-treated *huORFZ* embryos using FACS, followed by transplanting into SCI-adult zebrafish from transgenic line *huORFZ.* Cell transplantation followed the procedure described by Zeng *et al*. [56], except that four groups of donor cells were studied: (i) GFP(+) cells, (ii) GFP(+) cells close to rostral part of SCI site (rostral-GFP(+) cells), (iii) caudal-GFP(+) cells and (iv) GFAP(+) cells sorted from transgenic line *Tg(gfap:dTomato)* [63].

#### 4.13.1. Neuronal differentiation capability assay

The numbers of rostral-GFP(+) cells and caudal-GFP(+) cells differentiating into neurons *in vitro* were quantified by counting cells displaying neuron-like shape with an extended axon of at least 40 µm. We randomly chose a 2.25 cm^2^ area in the culture cell dish to count cells (*n* = 6 at each examined time point).

### 4.14. Neuronal extension measurements

We randomly selected 10 zebrafish from each group after lesser ablation of SrRCs at 54 hpi. Under 400-fold magnification of images (Zeiss LSM 780), we measured the length of axons using the algorithm in NeuronJ, which is an ImageJ software plugin, following the method described by Meijering *et al*. [64]. Briefly, each image was converted to an 8-bit tiff image, followed by tracing using the automated function in NeuronJ. The length of axons from rostral- and caudal-SrRCs was measured. In total, ten embryos were chosen to measure the length of extended axons, and an average was struck from these ten samples.

### 4.15. Statistical analysis

Unless otherwise indicated, each experiment was repeated at least three times. Animals were randomly assigned to different experimental groups, but no formal method of randomization was used. We used one-way ANOVA, followed by Dunn's multiple comparison test, or two-way ANOVA, followed by Student's *t*-test, for comparisons. Significance was determined at *p* value as indicated in the figure legends.

## Data accessibility

This article has no additional data.
